# The Retro-Buccal Method of Exposing the Third Branch of the Trigeminus

**Published:** 1896-06

**Authors:** 


### The Retro Buccal Method of Exposing the Third Branch of the Trigeminus.

The author reports two cases operated by this method.

The operation is not dangerous, is simple, and gives the least amount of interference with function. In a few days the patients were well, and only a very fine linear scar was left on the cheek; the facial nerve, Steno's duct and the muscles of mastication were not injured.

In the second case he succeeded, with the aid of Thiersch's nerve curling, in eliminating the infra-maxillary nerve, even in its intracranial course, through the Gasserian ganglion, together with a portion of the latter. Both operations illustrated the possibility of eliminating the trigeminal nerve, even intracranially, by an extracranial operation.—*Kronlein* (*Beitrage zur klin. Chir.*, XIV, No. 3, p. 725.)

This seems an uncertain and unscientific method of destroying the Gasserian ganglion. Even though it has been successful in these two instances there will undoubtedly be many others where the nerve will give way during the curling, leaving a well-defined stump, which, in all probability, will become the point of fixed pain, necessitating the Krause-Hartley operation finally. —*American Medico-Surgical Bulletin.*

